# Neck and Trunk Muscle Strength in Children With Spinal Muscular Atrophy Is Lower Than in Healthy Controls and Depends on Disease Type

**DOI:** 10.3389/fneur.2021.628414

**Published:** 2021-04-30

**Authors:** Agnieszka Stępień, Tomasz Osiak, Witold Rekowski, Andrzej Wit

**Affiliations:** ^1^Department of Rehabilitation, Józef Piłsudski University of Physical Education, Warsaw, Poland; ^2^ORTHOS Functional Rehabilitation Centre, Warsaw, Poland

**Keywords:** spinal muscular atrophy, muscle strength, trunk, neck, motor function, handheld dynamometry, physiotherapy

## Abstract

**Background:** Neck and trunk muscle strength and relationship with motor function in individuals with spinal muscular atrophy (SMA) is not investigated well. Information on maximum muscle strength that children with SMA may develop considerably expands the possibilities of assessing the effectiveness of pharmacological treatment methods and therapeutic procedures. This study sought to assess neck and trunk muscle strength in patients with SMA and to compare it with values noted in healthy children.

**Methods:** The study involved 56 individuals with SMA aged 5–16 not treated pharmacologically, including 9 patients with SMA type 1 (SMA1), 27 with SMA type 2 (SMA2), and 20 with SMA type 3 (SMA3). The control group included 111 healthy individuals aged 5–16. Neck and trunk muscle strength was assessed by means of a maximum voluntary isometric contraction method with the use of a handheld digital muscle tester MICROFET2. Moreover, relative strength was also calculated by standardising the maximum voluntary isometric contraction according to body mass. The Kruskal–Wallis test, Mann–Whitney *U*-test, and Spearman's rank correlation were used for statistical analysis.

**Results:** The reliability of the neck and trunk muscle strength measurements with the handheld digital muscle tester was excellent with ICC > 0.9. The values of muscle strength in SMA groups were significantly lower than in the control group. The values of relative torque of the neck muscles expressed in percentage values calculated with regard to the control group were at the level of 47.6–51.6% in SMA1 group, 54.8–58.1% in SMA2 group and 80.6–90.3% in SMA3 group. The percentage values for upper and lower trunk muscle strength were at the level of 42.6–68.4% in SMA1 group, 56.9–75.4% in SMA2 group and 76.7–94.8% in SMA3 group.

**Conclusion:** Handheld dynamometry provides reliable measures of neck and trunk muscle strength in SMA children. Neck and trunk muscle strength in children with SMA is lower than in healthy controls and depends on disease type, which confirms the theory based on clinical observations. Further, study is needed to investigate the effect of pharmacological treatment on the strength of the neck/trunk muscles, and relationship between neck and trunk muscle strength and motor capabilities.

## Introduction

Spinal muscular atrophy (SMA) is a rare neuromuscular disease involving anterior horn cells degeneration of lower motor neurons in the spinal cord caused by the loss of function mutations in the survival motor neuron 1 (SMN1) gene (1).

It is characterised by the fact that the first symptoms occur in different periods, there is a large variety of symptoms and there are different levels of intensity of motor disorders. The classification of SMA involves the time when the first symptoms occur and maximum motor capabilities (2, 3). The division that takes into account the functional state and is used while planning treatment procedures includes non-sitters, sitters, and walkers (4).

Muscle weakness is one of the typical symptoms of the disease. In the past, upper and lower limbs muscle strength in SMA patients was assessed with the use of both qualitative and quantitative methods (5–14). The weakening of proximal muscles compared to distal muscles and the weakening of lower limb muscles compared to upper limb muscles were noted (5, 7, 15). The correlations between the strength of selected muscles and motor functions in SMA patients have been analysed in various studies (8, 10, 11, 13–17). It has been revealed, inter alia, that the weakening of muscles occurs simultaneously with the deterioration of the patients' functional state (15) and increases with age (5, 9, 12, 14). Also, the correlations between the strength of particular muscles and physical activity have been analysed (10, 11, 16). Few projects have also demonstrated the reduced bite strength in patients with SMA (18, 19).

While focusing on neck and trunk muscle strength in SMA patients, it should be noted that there is a scarcity of studies on this issue. We can only cite the results of the research which revealed that improper head balance may negatively affect the function of swallowing (20). It was also proved that persons with SMA2 and SMA3 perform trunk movements in a sedentary position to a limited extent and with lower muscle activity than healthy individuals (21).

The above literature review enables us to conclude that there is a need for study focusing on assessing neck and trunk muscle strength in SMA patients. The neck and trunk muscles are active in many daily activities and their reduced muscle strength can affect quality of life. It is also important to note that assessing these groups of muscles may significantly support the interpretation of the effects of pharmacological treatment (3, 22, 23) and the evaluation of the effectiveness of the rehabilitation process as well as other therapeutic interventions.

The aim of the study conducted on a representative group was to reveal the values of the strength of neck and trunk muscles in children with different types of SMA as well as to compare them with the values of the strength of the same muscles in healthy children. Moreover, the assessment of the reliability of the measurements of neck and trunk muscle strength made with the use of a handheld muscle tester according to our own methodology was planned.

## Materials and Methods

### Participants

Children and adolescents aged 5–16 with genetically confirmed SMA, not treated with Nusinersen and not participating in clinical trials of other medications were qualified for the study. The type of SMA has been determined in the past by neurologists. Individuals using continuous tracheostomy ventilation, those after surgical treatment of scoliosis as well as those with severe spine and chest deformities that made it impossible to maintain a side lying position without support were excluded from the study. The study involved patients who used non-invasive ventilation, but were able to breathe independently. The control group included healthy persons aged 5–16.

The study was carried out during workshops organised by the SMA Foundation in Poland in the years 2017–2018 and during individual physiotherapy consultations. The main tests were preceded by the evaluation of the reliability of muscle strength measurements. The interobserver reliability assessment involved two trained physiotherapists (one with over 20 years and the other one with 1 year of professional experience) who independently measured strength in every participant with 2-hour intervals and recorded the results separately. The intraobserver measurements were performed twice by one researcher during individual consultations in a rehabilitation clinic with 1-hour intervals.

In total, muscle strength was tested in 56 SMA patients. The control group consisted of 111 healthy participants. Interobserver reliability assessment was carried out in a group of 31 individuals with SMA and intraobserver reliability was assessed in 20 SMA patients and 44 healthy participants.

Individuals qualified for the study as well as their legal guardians were informed about the aim of the study and planned procedures, and they gave their written consent. The study was accepted by the Senate Bioethical Committee (SKE 01-03/2017) and registered (ISRCTN63278972).

### Assessments

In the case of each SMA patient, their ability to turn from the supine position to the side lying position was assessed. The carers of SMA patients were asked to provide information on how often they help the child change the position at night.

**Figure 1:**
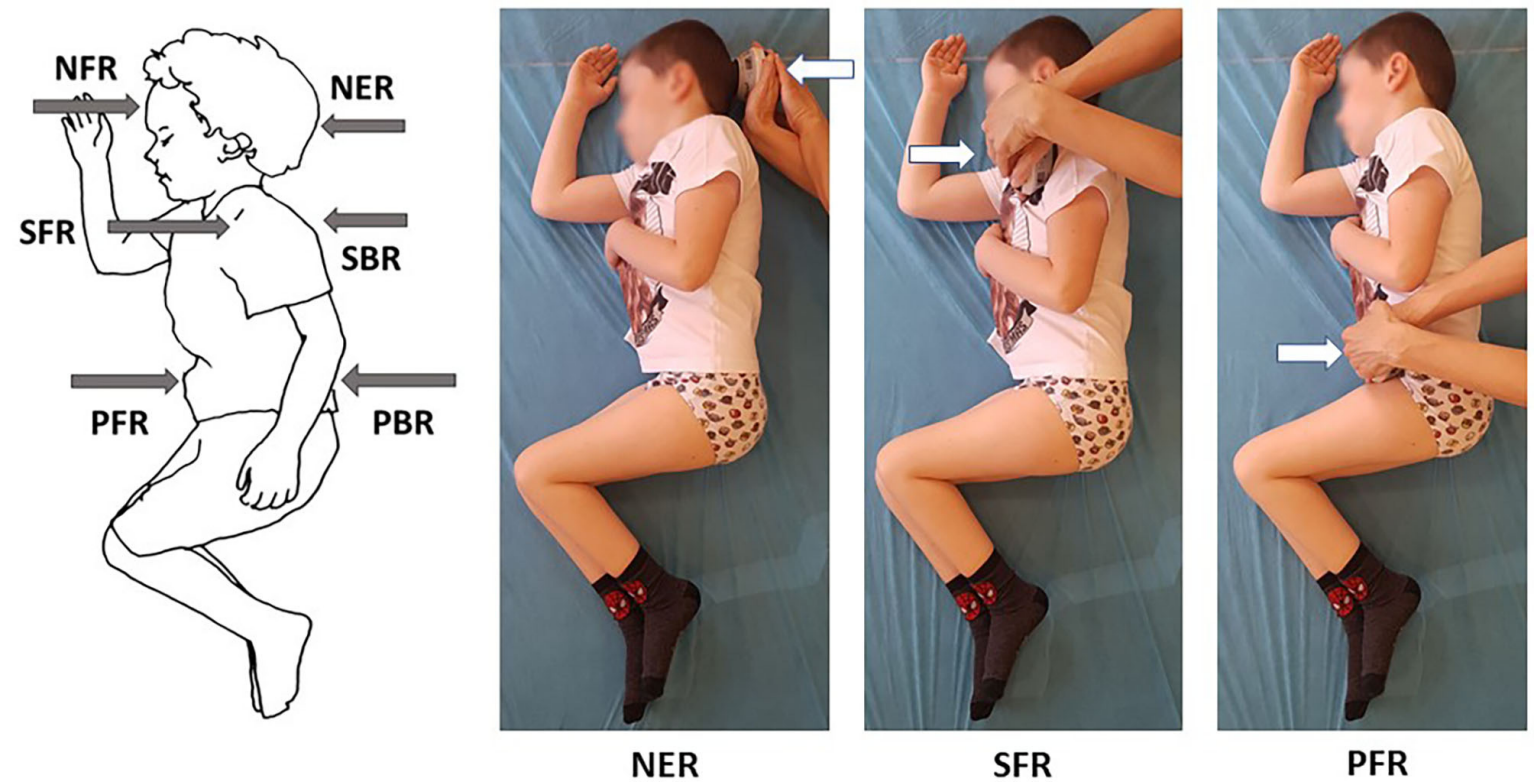
Selected measurements of neck and trunk muscle strength in side lying on the right in a 6-year-old with type 3 SMA.

Muscle strength examination was performed in a side lying position with hips bent at 45° and knees bent at 90° (Figure 1). Strength was measured by the examiner standing behind the participant with a handheld digital muscle tester MICROFET2, Hoggan Scientific LLC, which makes it possible to measure muscle strength in pounds, newtons, or kilogrammes with an accuracy of 0.1 pound, in the range of 0.8–300 pounds. The accuracy of the device is within 1%. The dynamometer has a calibration certificate. In the past it was revealed that the measurement made with this type of a dynamometer is a reliable form of muscle strength assessment (24, 25). To date, this device has not been used to assess neck and trunk muscle strength. Moreover, no research on children has been carried out.

The dynamometer was placed perpendicular to the head and trunk in the sagittal plane. The strength measurements were preceded by the linear measurements of the following distances of the examined individuals performed in a side lying position: (1) the middle of forehead — suprasternal notch, (2) the anterior part of the acromion of the side of the body higher above the surface — across the trunk toward the surface, (3) anterior superior iliac spine on the side of the body higher above the surface - across the pelvis toward the surface. These distances were treated as an arm of muscle activity in the area of head, upper trunk, and lower trunk.

The direct strength test involved 12 measurements of maximum voluntary isometric contraction, including 6 measurements on the left and 6 on the right side. Four measurements were made with the handheld tester placed on the head, i.e., centrally on the forehead above eyebrows (NFL — neck flexion in side lying on the left, NFR — neck flexion in side lying on the right) and at the back of the skull (NEL — neck extension in side lying on the left, NER — neck extension in side lying on the right). The strength of an upper part of the trunk was measured by placing the tester at the front of the acromion (SFL — right scapula forward in side lying on the left, SFR - left scapula forward in side lying on the right) and at the back on the lateral edge of the scapula near the spine of the scapula (SBL — right scapula backward in side lying on the left, SBR — left scapula backward in side lying on the right). The measurements of a lower part of the trunk were made with the tester held at the front of the pelvis near anterior superior iliac spine (PFL — right part of pelvis forward in side lying on the left, PFR — left part of pelvis forward in side lying on the right) and posterior superior iliac spine at the back of the pelvis (PBL — right part of pelvis backward in side lying on the left, PBR — left part of pelvis backward in side lying on the right) (Figure 1). Such a method of measuring the force arm and making measurements has not been presented in the literature so far. The methodology was adapted to the diverse condition of children with SMA.

Prior to the measurement, the participants were shown the device and the manner in which the measurement is performed. Also, instructions were provided and trial measurements (six different measurements on each side) performed as a warm-up. Moreover, the participants were asked to change their position actively during the trial measurements.

The main measurements were performed without trunk stabilisation in a previously determined order. The children were asked to maintain a side lying position resisting the force of the dynamometer with their head, arm, or pelvis. The participants were asked to refrain from head movements as well as upper and lower limb movements. The resistance was increased slowly while observing the participant's reactions. Each measurement lasted 10 s. The dynamometer registered the highest value obtained in newtons [N]. Due to the fact that fatigue might have occurred, each measurement was carried out once. If a simultaneous head or limbs movement occurred, the measurement was repeated.

The torque (T) was calculated by multiplying the force measurement (F) by the moment arm from the axis of motion (a) expressed in metres: T = F^*^a. The force measurements were normalised to body mass by dividing the value of the torque (T) by the participant's body mass (BM) expressed in kilogrammes: R = T/BM.

### Statistical Analysis

Statistical analyses were performed using the statistical software IBM SPSS Statistics version 20. First, the reliability of muscle strength measurements was assessed. To assess reliability, the interclass correlation coefficient (ICC) and 95% confidence intervals were applied (26). ICC values were interpreted in the following manner: below 0.40 — poor reliability, 0.40–0.59 — fair reliability, 0.60–0.74 — good reliability, 0.75–1.00 — excellent reliability (27).

Taking into account the lack of a normal distribution according to the Kolmogorov-Smirnov test, the statistical comparisons of data between groups were performed using the non-parametric Kruskal–Wallis test and the Mann–Whitney *U*-test.

Mean values and standard deviations of the tested parameters were calculated. Additionally, the percentage values of the mean measurements obtained in the SMA1, SMA2, and SMA3 groups were calculated in reference to the control group.

Spearman's rank correlation coefficient were used to analyse the correlations between the torque (T) measurements within SMA1, SMA2, SMA3, and control groups. Correlations between the age of the participants in the groups and the values of the strength measurements were also assessed. The following correlation interpretation was used: <0.3 — negligible correlation, 0.3–0.5 — low correlation, 0.5–0.7 — moderate correlation, 0.7–0.9 — high correlation, >0.9 — very high correlation (28). The level of significance was set at *p* ≤ 0.05.

## Results

### Interobserver and Intraobserver Reliability

The main study were preceded by an analysis of the reliability of muscle strength measurements. Interobserver reliability assessment was performed on a group of 31 individuals (13 girls and 18 boys) with SMA aged 5–16 (8.35 ± 3.44 years) with body mass of 25.13 ± 9.87 kg and body height of 1.27 ± 0.17 m. The examined group comprised three persons with SMA1, 20 with SMA2 and nine with SMA3. Eight out of nine participants with SMA3 were able to walk unassisted.

Intraobserver reliability was assessed by analysing 20 individuals with SMA (8 girls and 12 boys) aged 5–16 (8.16 ± 3.24 years) with body mass of 24.72 ± 10.36 kg and body height of 1.27 ± 0.18 m, as well as 44 healthy participants (21 girls and 23 boys) aged 5–16 (9.55 ± 2.84 years), with body mass of 32.79 ± 12.74 kg and body height of 1.39 ± 0.17 m. The group of SMA patients included 14 individuals with SMA2 and 6 with SMA3.

The results revealed excellent interobserver and intraobserver reliability for all the measurements (ICC > 0.9) expressed in the force [N] and T torque [Nm] values (Table 1). Interobsever reliability of the linear measurements was excellent with ICC = 0.941 (0.878–0.972) for the neck, ICC = 0.968 (0.933–0.984) for the upper trunk, and ICC = 0.967 (0.932–0.984).

**Table 1:** Interobserver and intraobserver reliability of the force F (N) and torque T (Nm) measurements.

**Measurements**	**Interobserver reliability** **(*****n*** **=** **31)**		**Intraobserver reliability** **(*****n*** **=** **64)**	
	**ICC**	**95% confidence interval**	**ICC**	**95% confidence interval**
NFL F [N]	0.978	0.953–0.989	0.986	0.976–0.991
NFL T [Nm]	0.975	0.948–0.988	0.990	0.984–0.994
NEL F [N]	0.986	0.971–0.993	0.985	0.976–0.991
NEL T [Nm]	0.991	0.982–0.996	0.990	0.984–0.994
NFR F [N]	0.976	0.950–0.988	0.988	0.980–0.992
NFR T [Nm]	0.984	0.967–0.992	0.992	0.987–0.995
NER F [N]	0.986	0.971–0.993	0.988	0.980–0.993
NER T [Nm]	0.987	0.973–0.994	0.992	0.987–0.995
SFL F [N]	0.964	0.926–0.983	0.983	0.971–0.990
SFLT [Nm]	0.979	0.956–0.990	0.990	0.984–0.984
SBL F [N]	0.964	0.925–0.983	0.992	0.987–0.995
SBL T [Nm]	0.977	0.951–0.989	0.995	0.992–0.997
SFR F [N]	0.971	0.940–0.986	0.976	0.960–0.985
SFR T [Nm]	0.978	0.955–0.990	0.986	0.976–0.991
SBR F [N]	0.956	0.908–0.979	0.992	0.986–0.995
SBR T [Nm]	0.955	0.907–0.978	0.995	0.992–0.997
PFL F [N]	0.987	0.973–0.984	0.981	0.968–0.988
PFL T [Nm]	0.988	0.975–0.994	0.989	0.981–0.993
PBL F [N]	0.969	0.936–0.985	0.984	0.974–0.990
PBL T [Nm]	0.976	0.949–0.988	0.991	0.985–0.995
PFR F [N]	0.975	0.949–0.988	0.982	0.971–0.989
PFR T [Nm]	0.976	0.950–0.988	0.990	0.984–0.994
PBR F [N]	0.979	0.956–0.990	0.959	0.933–0.975
PBR T [Nm]	0.977	0.952–0.989	0.973	0.956–0.984

*NFL, neck flexion in side lying on the left; NEL, neck extension in side lying on the left; NFR, neck flexion in side lying on the right; NER, neck extension in side lying on the right; SFL, right scapula forward in side lying on the left; SBL, right scapula backward in side lying on the left; SFR, left scapula forward in side lying on the right; PFL, right part of pelvis forward in side lying on the left; PFR, left part of pelvis forward in side lying on the right; PBL, right part of pelvis backward in side lying on the left; PBR, left part of pelvis backward in side lying on the right; N, newton; m, metre; n, number of participants*.

### Characteristics of the Participants

There were 25 girls and 31 boys in the group of 56 SMA participants (mean age 7.9 ± 2.6 years, body mass 23.0 ± 8.9 kg, body height 1.22 ± 0.15 m). The SMA group comprised 9 SMA1 patients, 27 patients with SMA2, as well as 20 children with SMA3. The SMA group included 9 SMA1 non-sitters, 2 SMA2 non-sitters, 25 SMA2 sitters, 5 SMA3 sitters (non-ambulant) and 15 SMA3 ambulant patients. The control group consisted of 111 healthy participants (Table 2).

**Table 2:** Characteristic of participants aged 5–16.

	**SMA1 (*n* = 9)**	**SMA2 (*n* = 27)**	**SMA3 (*n* = 20)**	**Control (*n* = 111)**
**General information**				
Age [years]	7.89 ± 2.39	8.28 ± 2.87	7.52 ± 2.34^*^	8.89 ± 2.44
Body mass [kg]	21.67 ± 6.65^**^	24.37 ± 9.08^**^	21.80 ± 9.57^***^	31.60 ± 12.09
Percentile body mass	18.67 ± 25.97 (3–75)	23.37 ± 25.17 (0–75)	23.45 ± 23.93 (0–75)	45.8 ± 24.5 (3–90)
Body height [m]	1.21 ± 0.12^**^	1.25 ± 0.14^**^	1.17 ± 0.15^***^	1.37 ± 0.17
Percentile body height	16.56 ± 19.24 (3–50)	21.81 ± 21.99 (0–75)	10.85 ± 13.72 (0–75)	60.9 ± 24.0 (3–95)
Gender	3 girls, 6 boys	13 girls, 14 boys	9 girls, 11 boys	52 girls, 59 boys
Non-sitters [*n*]	9 [100.0%]	2 [7.4%]	0 [0.0%]	0 [0.0%]
Sitters [*n*]	0 [0.0%]	25 [92.6%]	5 [25.0%]	0 [0.0%]
Walkers [*n*]	0 [0.0%]	0 [0.0%]	15 [75.0%]	111 [100.0%]
Ability to turn to side [*n*]	0 [0.0%]	16 [59.3%]	20 [100.0%]	111 [100.0%]
Help needed at night [*n*]	9 [100.0%]	24 [88.9%]	5 [25.0%]	0 [0.0%]
Scoliosis [*n*/%]	8 [88.9%]	19 [70.4%]	8 [40.0%]	0 [0.0%]
**Mean values (**±**SD) of T and R**				
NFL T [Nm]	3.29 ± 1.37^***^	4.08 ± 1.90^***^	5.85 ± 2.85^***^	9.59 ± 3.79
NFL R [Nm/kg]	0.16 ± 0.07^***^	0.18 ± 0.07^***^	0.28 ± 0.09	0.31 ± 0.07
NFR T [Nm]	3.31 ± 1.38^***^	3.86 ± 1.74^***^	5.35 ± 3.01^***^	9.55 ± 3.73
NFR R [Nm/kg]	0.16 ± 0.08^***^	0.17 ± 0.06^***^	0.25 ± 0.07^**^	0.31 ± 0.07
NEL T [Nm]	4.34 ± 1.30^***^	5.61 ± 2.81^***^	7.87 ± 4.21^***^	13.20 ± 5.21
NEL R [Nm/kg]	0.21 ± 0.07^***^	0.24 ± 0.10^***^	0.37 ± 0.11^*^	0.43 ± 0.11
NER T [Nm]	4.19 ± 1.33^***^	5.63 ± 2.95^***^	8.06 ± 3.89^***^	13.16 ± 5.29
NER R [Nm/kg]	0.20 ± 0.07^***^	0.24 ± 0.11^***^	0.37 ± 0.09^*^	0.42 ± 0.11
SFL T [Nm]	4.41 ± 1.65^***^	5.80 ± 2.45^***^	7.21 ± 3.87^***^	13.38 ± 6.13
SFL R [Nm/kg]	0.22 ± 0.09^***^	0.25 ± 0.10^***^	0.33 ± 0.06^***^	0.43 ± 0.11
SFR T [Nm]	4.17 ± 1.72^***^	5.67 ± 2.78^***^	7.16 ± 3.36^***^	13.71 ± 6.22
SFR R [Nm/kg]	0.20 ± 0.08^***^	0.25 ± 0.11^***^	0.34 ± 0.09^***^	0.43 ± 0.10
SBL T [Nm]	6.65 ± 3.65^***^	8.54 ± 3.77^***^	10.21 ± 4.22^***^	17.34 ± 7.12
SBL R [Nm/kg]	0.32 ± 0.19^***^	0.37 ± 0.16^***^	0.48 ± 0.12^*^	0.56 ± 0.13
SBR T [Nm]	5.94 ± 2.68^***^	7.66 ± 3.66^***^	10.93 ± 4.31^***^	17.96 ± 7.67
SBR R [Nm/kg]	0.28 ± 0.13^***^	0.33 ± 0.15^***^	0.52 ± 0.14	0.58 ± 0.15
PFL T [Nm]	4.29 ± 1.84^***^	6.57 ± 3.53^***^	8.43 ± 4.79^***^	14.96 ± 7.05
PFL R [Nm/kg]	0.20 ± 0.09^***^	0.28 ± 0.13^***^	0.38 ± 0.12^***^	0.47 ± 0.11
PFR T [Nm]	4.66 ± 1.50^***^	6.70 ± 4.70^***^	8.18 ± 4.05^***^	15.19 ± 6.88
PFR R [Nm/kg]	0.22 ± 0.07^***^	0.29 ± 0.21^***^	0.38 ± 0.11^***^	0.48 ± 0.11
PBL T [Nm]	8.20 ± 4.33^***^	9.95 ± 4.66^***^	11.14 ± 3.88^***^	17.15 ± 5.85
PBL R [Nm/kg]	0.39 ± 0.22^***^	0.43 ± 0.17^***^	0.54 ± 0.17	0.57 ± 0.15
PBR T [Nm]	7.88 ± 3.37^***^	9.22 ± 4.22^***^	11.63 ± 5.31^***^	17.51 ± 6.22
PBR R [Nm/kg]	0.38 ± 0.17^***^	0.39 ± 0.16^***^	0.55 ± 0.16	0.58 ± 0.15

*Mean values (±SD) of the torque (T) and relative torque (R) in SMA1, SMA2, SMA3, and control groups. Comparison of the group SMA1, SMA2, and SMA3 with the control group*.

*NFL, neck flexion in side lying on the left; NEL, neck extension in side lying on the left; NFR, neck flexion in side lying on the right; NER, neck extension in side lying on the right; SFL, right scapula forward in side lying on the left; SBL, right scapula backward in side lying on the left; SFR, left scapula forward in side lying on the right; PFL, right part of pelvis forward in side lying on the left; PFR, left part of pelvis forward in side lying on the right; PBL, right part of pelvis backward in side lying on the left; PBR, left part of pelvis backward in side lying on the right; T, the torque; R, the relative torque; N, newton; m, metre; kg, kilogramme; n, number of participants; SMA, spinal muscular atrophy*.

*Significance of differences in relation to the control group:*

* *at the level of 0.05 > p > 0.01*,

** *at the level of 0.01 ≥ p ≥ 0.001*,

*** *at the level of p < 0.001*.

Children with SMA1, SMA2, SMA3, and healthy controls did not differ in terms of age in the analysis using the Kruskal Wallis test, but comparisons between groups with the Mann–Whitney *U*-test demonstrated a difference between the SMA3 and control groups. Significant differences regarding body height and body mass were noted between the control group and SMA1, SMA2, and SMA3 groups (*p* < 0.05). SMA1, SMA2, and SMA3 groups did not differ in terms of age, body height and mass (*p* > 0.05).

### Functional State of SMA Patients

Nearly 65% of the study participants with SMA were able to turn to the left and to the right side unassisted, while the remaining patients (9 SMA1, 11 SMA2) encountered problems performing this activity in a supine position. The carers' help at night was necessary in the case of 68% of the participants, while 32% of the patients (3 SMA2, 15 SMA3) changed the position without assistance (Table 2).

The guardians of children with SMA1 declared that they helped their children an average of 4.6 ± 1.6 times every night, in the case of children with SMA 2 it was 2.6 ± 1.5 times, while in the SMA3 group parents got up 0.5 ± 1.0 times per night. Children with SMA1 significantly more often needed help at night while changing a position compared to SMA2 (*p* < 0.01), SMA3 (*p* < 0.001), and control group (*p* < 0.001). In turn, children with SMA2 needed help more often than the participants from SMA3 and control group (*p* < 0.001). Moreover, differences in the frequency of helping children were noted between SMA3 group and control group (*p* < 0.001).

### Neck Muscle Strength in SMA and Control Groups

Table 2 includes the values of the torque (T) and relative torque (R) of the neck flexors and extensors and trunk muscles in SMA and control groups.

Absolute torque (T) in the neck area in the control group was always significantly higher than the values obtained by patients with SMA1, SMA2, and SMA3 (Table 2). Children with SMA3 were significantly stronger than SMA1 and SMA2 participants.

A larger number of differences between the groups were noted after taking into account body mass of the participants (R coefficient). All the values of R coefficient obtained by individuals with SMA1 SMA2 were significantly lower than in the control group. The results of the measurements in SMA3 group were similar to the control group only in the measurement of neck flexion in side lying on the left (NFL) (Table 2). The values of the measurements in SMA1 and SMA2 groups proved to be significantly lower than in SMA3 group (*p* < 0.05). No significant differences were noted between the values of the strength of neck muscles in SMA1 and SMA2 groups.

**Figure 2:**
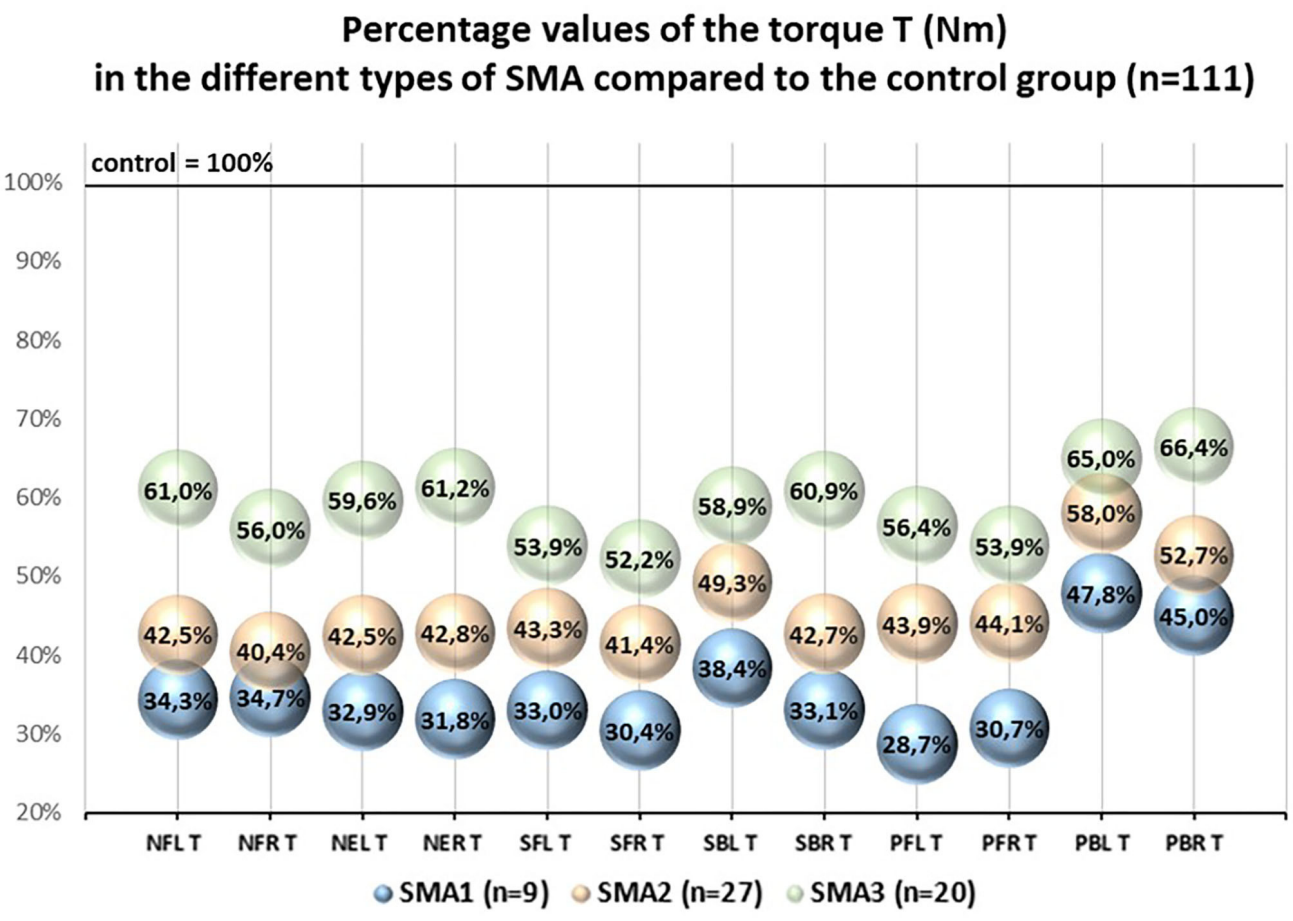
Pecentage values of the torque T (Nm) in the different types of SMA compared to the control group (*n* = 111).

**Figure 3:**
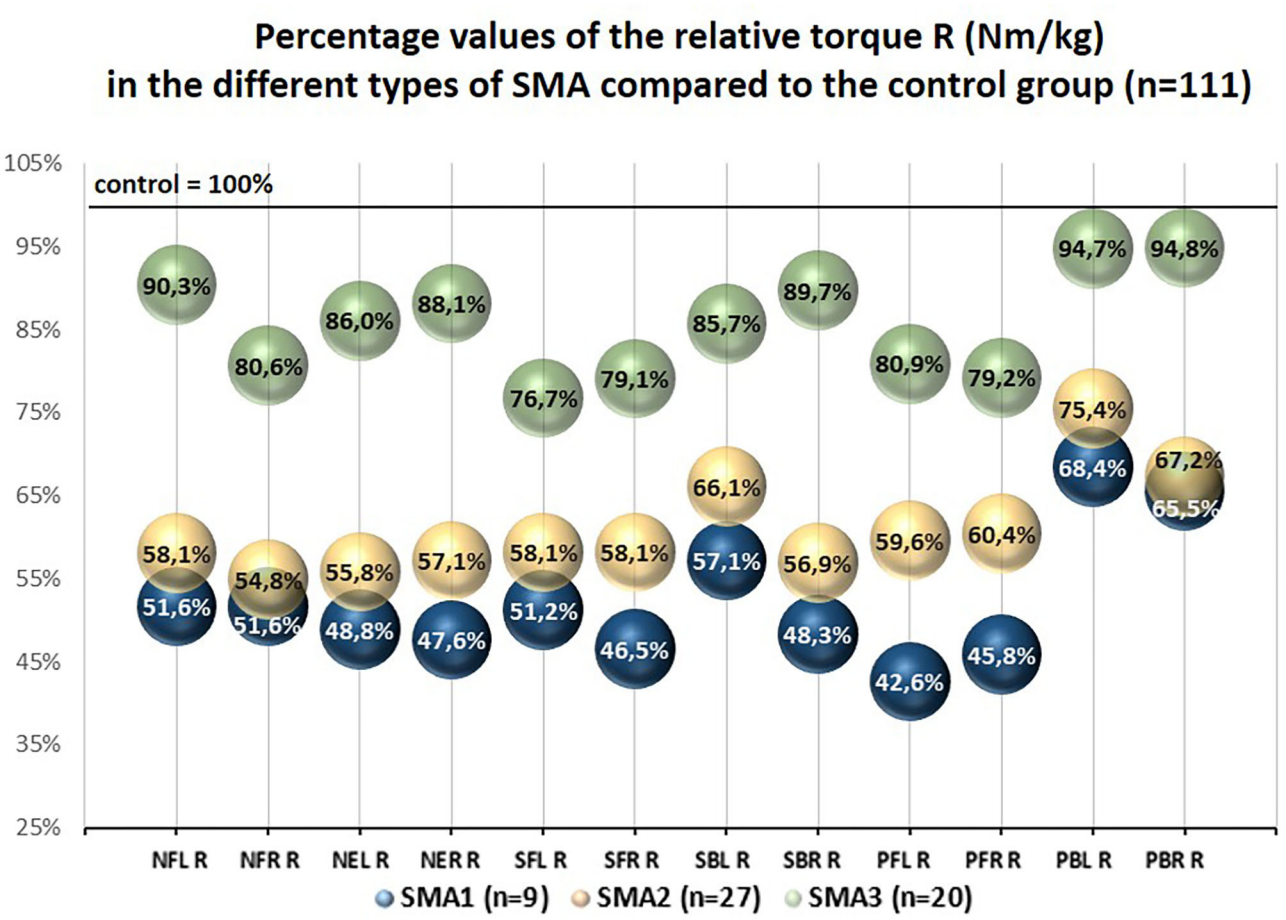
Percentage values of the relative torque R (Nm/kg) in the different types of SMA compared to the control group (*n* = 111).

The percentage values of the examined T and R parameters with regard to the control group treated as 100% were different in SMA1, SMA2, and SMA3 groups. The lowest percentage values were noted in SMA1 group, medium ones in SMA2 group, while the highest ones were noted in SMA3 group (Figures 2, 3).

Relative torques in neck muscles in children with SMA who could turn to the left and right side unassisted were significantly higher (*p* < 0.001) compared to children who needed help while turning from a supine position to side lying.

### Trunk Muscle Strength in SMA and Control Groups

The comparative analysis of the results of measurements made in the area of upper and lower trunk revealed a number of significant differences between the control group and individuals from groups SMA1, SMA2, and SMA3. Healthy children achieved significantly higher values of torque T compared to children with SMA1, SMA2, and SMA3 (Table 2). No significant differences were found between groups SMA1 and SMA2 regarding torques (T). There were also no significant differences between the SBL and PBL values in the SMA1 and SMA3 groups, as well as between the values of the torque T for the SFL, SBL, PFL, PBL, SFR, PFR measurements in the SMA2, and SMA3 groups.

The values of the relative torque (R) in SMA1 and SMA2 group were significantly lower than in the control group (*p* < 0.001). In SMA3 group the value of R coefficient did not differ significantly from the control group as far as SBR, PBL, and PBR parameters are concerned. It means that taking into account body mass, the strength of muscles responsible for trunk movement to the back was similar in SMA3 patients and in healthy individuals. Simultaneously, muscles responsible for trunk movement to the front were weaker than the norm. The R coefficient had a higher value in SMA3 group compared to SMA1 in all the measurements apart from the PBL measurement. The SMA2 patients achieved significantly lower values of R coefficient in all measurements than SMA3 group (Table 2). The SMA1 and SMA2 type participants achieved similar R-values in all measurements.

The percentage values of absolute torques (T) of upper and lower trunk muscles obtained in SMA groups ranged from 28.7 to 66.4% with regard to the control group, while the values of relative torque (R) ranged from 42.6 to 94.8% and were closely related to the group of muscles under investigation and SMA type (Figures 2, 3).

In the case of children with SMA who were able to turn unassisted from the supine position to the left or right side, significantly higher values of R coefficient were noted for the trunk measurements compared to children who were unable to turn (*p* < 0.05).

### Correlations Between the Torque (T) Measurements Within SMA1, SMA2, SMA3 Groups

In the case of SMA2 and SMA3 patients, positive correlations between the majority of the measurements in the neck, upper trunk and lower trunk areas were noted, similar to the control group, where strong positive correlations were observed between the pairs of all measurements with the correlation coefficient of 0.745 ≤ *r* ≥ 0.979. In the SMA2 participants, all correlations were statistically significant, as in the control group. No significant correlation was observed between the values of the neck flexors strength and some measurements in the upper and lower trunk within SMA3 group (Table 3).

**Table 3:** The Spearman rank correlation coefficient (r) for the T torque values and age within SMA1, SMA2, SMA3, and control groups aged 5–16.

	**NFL** **r**	**NEL** ** r**	**SFL** **r**	**SBL** **r**	**PFL** **r**	**PBL** ** r**	**NFR** **r**	**NER** ** r**	**SFR** **r**	**SBR** ** r**	**PFR** **r**	**PBR** **r**
**SMA1 (*****n*** **=** **9)**												
NFL	-	0.533	717^*^	0.767^*^	0.783^*^	0.733^*^	0.733^*^	0.417	0.435	0.433	0.800^*^	0.550
NEL	0.533	-	0.850^**^	0.750^*^	0.717^*^	0.933^***^	0.800^*^	0.883^**^	0.753^*^	0.767^*^	0.533	0.883^**^
SFL	0.717^*^	0.850^**^	-	0.800^*^	0.650	0.833^**^	0.983^***^	0.767^*^	0.410	0.617	0.550	0.683^*^
SBL	0.767^*^	0.750^*^	0.800^*^	-	0.633	0.883^**^	0.783^*^	0.500	0.510	0.583	0.650	0.717^*^
PFL	0.783^*^	0.717^*^	0.650	0.633	-	0.850^**^	0.617	0.767^*^	0.828^**^	0.800^*^	0.933^***^	0.833^**^
PBL	0.733^*^	0.933^***^	0.833^**^	0.883^**^	0.850^**^	-	0.800^*^	0.800^*^	0.795^*^	0.817^**^	0.750^*^	0.933^**^
NFR	0.733^*^	0.800^*^	0.983^***^	0.783^*^	0.617	0.800^*^	-	0.700^*^	0.326	0.583	0.533	0.667^*^
NER	0.417	0.883^**^	0.767^*^	0.500	0.767^*^	0.800^*^	0.700^*^	-	0.736^*^	0.867^**^	0.583	0.833^**^
SFR	0.435	0.753^*^	0.410	0.510	0.828^**^	0.795^*^	0.326	0.736^*^	-	0.762^*^	0.686^*^	0.837^**^
SBR	0.433	0.767^*^	0.617	0.583	0.800^*^	0.817^**^	0.583	0.867^**^	0.762^*^	-	0.750^*^	0.933^***^
PFR	0.800^*^	0.533	0.550	0.650	0.933^***^	0.750^**^	0.533	0.583	0.686^*^	0.770^*^	-	0.733^*^
PBR	0.550	0.883^**^	0.683^*^	0.717^*^	0.833^**^	0.933^**^	0.667^*^	0.833^**^	0.837^**^	0.933^***^	0.733^*^	-
Age	0.622	0.521	0.286	0.437	0.866^**^	0.681^*^	0.269	0.513	0.857^**^	0.655	0.824^**^	0.740^*^
**SMA2 (*****n*** **=** **27)**												
NFL	-	0.646^***^	0.826^***^	0.645^***^	0.504^**^	0.690^***^	0.901^***^	0.597^**^	0.741^***^	0.505^**^	0.588^**^	0.625^***^
NEL	0.646^***^	-	0.746^***^	0.706^***^	0.574^**^	0.665^**^	0.604^**^	0.880^***^	0.690^***^	0.747^***^	0.642^***^	0.672^***^
SFL	0.826^***^	0.746^***^	-	0.632^***^	0.662^***^	0.651^***^	0.739^***^	0.714^***^	0.798^***^	0.474^*^	0.629^***^	0.665^***^
SBL	0.645^***^	0.706^***^	0.632^***^	-	0.578^**^	0.788^***^	0.618^**^	0.615^**^	0.573^**^	0.719^***^	0.775^***^	0.779^***^
PFL	0.504^**^	0.574^**^	0.662^***^	0.578^**^	-	0.607^**^	0.548^**^	0.531^**^	0.590^**^	0.599^**^	0.805^***^	0.658^***^
PBL	0.690^***^	0.665^**^	0.651^***^	0.788^***^	0.607^**^	-	0.671^**^	0.566^**^	0.628^***^	0.689^***^	0.786^***^	0.880^***^
NFR	0.901^***^	0.604^**^	0.739^***^	0.618^**^	0.548^**^	0.671^***^	-	0.580^**^	0.712^***^	0.559^**^	0.630^***^	0.585^**^
NER	0.597^**^	0.880^***^	0.714^***^	0.615^**^	0.531^**^	0.566^**^	0.580^**^	-	0.747^***^	0.673^***^	0.466^**^	0.588^**^
SFR	0.741^***^	0.690^***^	0.798^***^	0.573^**^	0.590^**^	0.628^***^	0.712^***^	0.749^***^	-	0.569^**^	0.568^**^	0.576^**^
SBR	0.505^**^	0.747^***^	0.474^*^	0.719^***^	0.599^**^	0.689^***^	0.559^**^	0.673^***^	0.569^**^	-	0.672^***^	0.748^***^
PFR	0.588^**^	0.642^***^	0.629^***^	0.775^***^	0.805^***^	0.786^***^	0.630^***^	0.446^*^	0.568^**^	0.672^***^	-	0.766^***^
PBR	0.625^***^	0.672^***^	0.665^***^	0.779^***^	0.658^***^	0.880^***^	0.585^**^	0.588^**^	0.576^**^	0.748^***^	0.766^***^	-
Age	0.356	0.474^*^	0.498^**^	0.351	0.525^**^	0.393^*^	0.337	0.269	0.397^*^	0.294	0.629^***^	0.557^**^
**SMA3 (*****n*** **=** **20)**												
NFL	-	0.475^*^	0.633^**^	0.332	0.217	0.180	0.737^***^	0.542^*^	0.471^*^	0.245	0.330	0.300
NEL	0.475^*^	-	0.774^***^	0.743^***^	0.574^**^	0.725^***^	0.636^**^	0.836^***^	0.729^***^	0.797^***^	0.683^***^	0.753^***^
SFL	0.633^**^	0.774^***^	-	0.800^***^	0.693^**^	0.696^**^	0.704^**^	0.859^**^	0.830^**^	0.729^**^	0.672^**^	0.798^***^
SBL	0.332	0.743^***^	0.800^***^	-	0.758^***^	0.859^***^	0.471^*^	0.788^***^	0.795^***^	0.911^***^	0.809^***^	0.788^***^
PFL	0.217	0.574^**^	0.693^**^	0.758^***^	-	0.713^***^	0.495^*^	0.695^**^	0.630^**^	0.693^**^	0.719^***^	0.830^***^
PBL	0.180	0.725^***^	0.696^**^	0.859^***^	0.713^***^	-	0.389	0.659^**^	0.788^***^	0.883^***^	0.743^***^	0.842^***^
NFR	0.737^***^	0.636^**^	0.704^**^	0.471^*^	0.495^*^	0.389	-	0.660^**^	0.583^**^	0.403	0.411	0.561^*^
NER	0.542^*^	0.836^***^	0.859^**^	0.788^***^	0.695^**^	0.659^**^	0.660^**^	-	0.714^***^	0.707^***^	0.544^*^	0.707^***^
SFR	0.471^*^	0.729^***^	0.830^**^	0.795^***^	0.630^**^	0.788^***^	0.583^**^	0.714^***^	-	0.786^***^	0.638^**^	0.701^**^
SBR	0.245	0.729^***^	0.729^**^	0.911^***^	0.693^**^	0.883^***^	0.403	0.707^***^	0.786^***^	-	0.862^***^	0.832^***^
PFR	0.330	0.683^***^	0.672^**^	0.809^***^	0.719^***^	0.743^***^	0.411	0.544^*^	0.638^**^	0.862^***^	-	0.798^***^
PBR	0.300	0.753^***^	0.798^***^	0.788^***^	0.830^***^	0.842^***^	0.561^*^	0.707^***^	0.701^**^	0.832^***^	0.798^***^	-
Age	0.508^*^	0.567^**^	0.711^***^	0.657^**^	0.734^***^	0.547^*^	0.538^*^	0.694^**^	0.779^***^	0.609^**^	0.652^**^	0.625^**^
**CONTROL (*****n*** **=** **111)**												
NFL	-	0.862^***^	0.849^***^	0.835^***^	0.853^***^	0.768^***^	0.979^***^	0.864^***^	0.864^***^	0.830^***^	0.829^***^	0.756^***^
NEL	0.862^***^	-	0.827^***^	0.899^***^	0.824^***^	0.819^***^	0.876^***^	0.970^***^	0.846^***^	0.904^***^	0.801^***^	0.837^***^
SFL	0.849^***^	0.827^***^	-	0.833^***^	0.837^***^	0.791^***^	0.852^***^	0.824^***^	0.900^***^	0.823^***^	0.841^***^	0.801^***^
SBL	0.835^***^	0.899^***^	0.833^***^	-	0.861^***^	0.872^***^	0.841^***^	0.910^***^	0.851^***^	0.944^***^	0.849^***^	0.856^***^
PFL	0.853^***^	0.824^***^	0.837^***^	0.861^***^	-	0.824^***^	0.858^***^	0.854^***^	0.865^***^	0.856^***^	0.916^***^	0.832^***^
PBL	0.768^***^	0.819^***^	0.791^***^	0.872^***^	0.824^***^	-	0.770^***^	0.843^***^	0.745^***^	0.865^***^	0.808^***^	0.922^***^
NFR	0.979^***^	0.876^***^	0.852^***^	0.841^***^	0.858^***^	0.770^***^	-	0.873^***^	0.869^***^	0.843^***^	0.832^***^	0.778^***^
NER	0.864^***^	0.970^***^	0.824^***^	0.910^***^	0.854^***^	0.843^***^	0.873^***^	-	0.842^***^	0.906^***^	0.816^***^	0.848^***^
SFR	0.864^***^	0.846^***^	0.900^***^	0.851^***^	0.865^***^	0.745^***^	0.869^***^	0.842^***^	-	0.848^***^	0.863^***^	0.769^***^
SBR	0.830^***^	0.904^***^	0.823^***^	0.944^***^	0.856^***^	0.865^***^	0.843^***^	0.906^***^	0.848^***^	-	0.823^***^	0.887^***^
PFR	0.829^***^	0.801^***^	0.841^***^	0.849^***^	0.916^***^	0.808^***^	0.832^***^	0.816^***^	0.863^***^	0.823^***^	-	0.803^***^
PBR	0.756^***^	0.837^***^	0.801^***^	0.856^***^	0.832^***^	0.922^***^	0.778^***^	0.848^***^	0.769^***^	0.887^***^	0.803^***^	-
Age	0.819^***^	0.762^***^	0.792^***^	0.799^***^	0.866^***^	0.714^***^	0.818^***^	0.790^***^	0.826^***^	0.764^***^	0.836^***^	0.715^***^

*NFL, neck flexion in side lying on the left; NEL, neck extension in side lying on the left; NFR, neck flexion in side lying on the right; NER, neck extension in side lying on the right; SFL, right scapula forward in side lying on the left; SBL, right scapula backward in side lying on the left; SFR, left scapula forward in side lying on the right; PFL, right part of pelvis forward in side lying on the left; PFR, left part of pelvis forward in side lying on the right; PBL, right part of pelvis backward in side lying on the left; PBR, left part of pelvis backward in side lying on the right; n, number of participants. Significance level:*

* *0.05 > p ≥ 0.01*,

** *0.01 > p ≥ 0.001*,

*** *< 0.001*.

In SMA1 group, the correlation between the pairs of measurements was not always significant (Table 3). No significant correlations were noted in this group between several pairs of measurements of head — scapula, scapula — scapula, scapula — pelvis. Therefore, it has to be concluded that an increase in the values of the measurements in one body part in SMA patients does not always mean an increase in other body parts, as in the control group. The highest variability of absolute torque was observed in the SMA1 group.

In the SMA3 and control groups, a significant moderate or high positive correlation was observed between the increase in all values of the T torque and the age of the participants. In the SMA1 and SMA2 groups, only some strength measurements showed a significant correlation with age, which may indicate a decrease in muscle strength with age in these groups.

### Neck and Trunk Muscle Strength in SMA3 and Healthy Participants Aged 5–10 Years

Due to several factors that may affect the results of statistical analysis, such as, different age and functional status, possible influence of growth, and puberty on the development of skeletal deformities, changes in the structure and function of muscles resulting from the progression of the disease, all participants with type 3 SMA and healthy children aged 5–10 years were separated from the study group as a new group. The SMA3 participants (*n* = 18; 4 sitters, 14 ambulant) and controls (*n* = 77) did not differ in term of age, but participants with SMA3 had lower values of height and weight. All children from both subgroups were able to turn sideways on their own, but children with SMA3 used their parents' help at night significantly more often (Table 4). The analysis showed significant differences between all T torque values and the majority values of R coefficients obtained in the SMA3 participants and control group (Table 4).

**Table 4:** Characteristic of SMA3 and healthy participants aged 5–10.

	**SMA 3 aged 5–10 (*n* = 18)**	**SMA3 ambulant aged 5–10 (*n* = 14)**	**Control aged 5–10 (*n* = 77)**
**General information**			
Age [years]	6.92 ± 1.38	6.79 ± 1.42	7.66 ± 1.69
Body mass [kg]	19.72 ± 5.93^**^	20.00 ± 6.34^*^	25.34 ± 6.88
Percentile body mass	21.33 ± 25.70 (0–75)	23.64 ± 28.17 (0–75)	38.73 ± 22.14 (3–90)
Body height [m]	114.06 ± 10.99^***^	113.71 ± 11.61^***^	127.90 ± 11.45
Percentile body height	12.33 ± 14.59 (0–50)	13.14 ± 15.70 (3–50)	53.72 ± 24.30 (3–95)
Gender	9 girls, 9 boys	8 girls, 6 boys	38 girls, 39 boys
Sitters [*n*]	4 [22.2%]	0 [0%]	0 [0%]
Walkers [*n*]	14 [77.8%]	14 [100%]	77 [100%]
Ability to turn to side [*n*]	18 [100%]	14 [100%]	77 [100%]
Help needed at night [*n*]	5 [27.8%]	3 [21.4%]	0 [0%]
Scoliosis [*n*/%]	7 [38.9%]	4 [28.6%]	0 [0%]
**Mean values (**± **SD) of T and R**			
NFL T [Nm]	5.15 ± 1.80^***^	4.97 ± 1.98^***^	7.78 ± 2.13
NFL R [Nm/kg]	0.27 ± 0.10	0.26 ± 0.10	0.31 ± 0.07
NFR T [Nm]	4.53 ± 1.41^***^	4.27 ± 1.40^***^	7.85 ± 2.25
NFR R [Nm/kg]	0.24 ± 0.07^***^	0.22 ± 0.07^***^	0.32 ± 0.07
NEL T [Nm]	6.83 ± 2.60^***^	6.84 ± 2.97^***^	11.01 ± 3.77
NEL R [Nm/kg]	0.36 ± 0.11^*^	0.35 ± 0.11^*^	0.44 ± 0.11
NER T [Nm]	7.13 ± 2.55^***^	7.13 ± 2.91^***^	10.74 ± 3.57
NER R [Nm/kg]	0.37 ± 0.09^*^	0.36 ± 0.09^*^	0.43 ± 0.11
SFL T [Nm]	6.29 ± 1.82^***^	6.22 ± 1.89^***^	10.61 ± 3.44
SFL R [Nm/kg]	0.33 ± 0.06^***^	0.32 ± 0.07^***^	0.42 ± 0.11
SFR T [Nm]	6.37 ± 2.07^***^	6.22 ± 1.93^***^	10.83 ± 3.47
SFR R [Nm/kg]	0.33 ± 0.09^***^	0.32 ± 0.08^***^	0.43 ± 0.10
SBL T [Nm]	9.31 ± 3.06^***^	9.46 ± 3.45^***^	14.26 ± 4.71
SBL R [Nm/kg]	0.48 ± 0.13^*^	0.48 ± 0.12^*^	0.57 ± 0.13
SBR T [Nm]	9.95 ± 3.05^***^	9.99 ± 3.42^**^	14.76 ± 4.81
SBR R [Nm/kg]	0.52 ± 0.14	0.52 ± 0.14	0.58 ± 0.15
PFL T [Nm]	7.54 ± 3.88^***^	7.97 ± 4.25^**^	11.74 ± 4.43
PFL R [Nm/kg]	0.40 ± 0.12^**^	0.39 ± 0.13^*^	0.46 ± 0.11
PFR T [Nm]	7.36 ± 3.23^***^	7.55 ± 3.63^***^	12.18 ± 4.58
PFR R [Nm/kg]	0.38 ± 0.11^**^	0.38 ± 0.12^*^	0.48 ± 0.12
PBL T [Nm]	10.54 ± 3.28^***^	10.73 ± 3.62^**^	14.96 ± 4.28
PBL R [Nm/kg]	0.56 ± 0.17	0.56 ± 0.17	0.60 ± 0.15
PBR T [Nm]	10.43 ± 3.12^***^	10.68 ± 3.40^**^	15.14 ± 4.43
PBR R [Nm/kg]	0.55 ± 0.17	0.56 ± 0.19	0.61 ± 0.10

*Mean values (±SD) of the torque (T) and relative torque (R) in SMA3, SMA3 ambulant, and control groups. Comparison of the group SMA3 and SMA3 ambulant with the control group*.

*NFL, neck flexion in side lying on the left; NEL, neck extension in side lying on the left; NFR, neck flexion in side lying on the right; NER, neck extension in side lying on the right; SFL, right scapula forward in side lying on the left; SBL, right scapula backward in side lying on the left; SFR, left scapula forward in side lying on the right; PFL, right part of pelvis forward in side lying on the left; PFR, left part of pelvis forward in side lying on the right; PBL, right part of pelvis backward in side lying on the left; PBR, left part of pelvis backward in side lying on the right; T, the torque; R, the relative torque; N, newton; m, metre; kg, kilogramme; n, number of participants; SMA, spinal muscular atrophy. Significance of differences in relation to the control group*:

* *at the level of 0.05 > p > 0.01*,

** *at the level of 0.01 ≥ p ≥ 0.001*,

*** *at the level of p < 0.001*.

**Figure 4:**
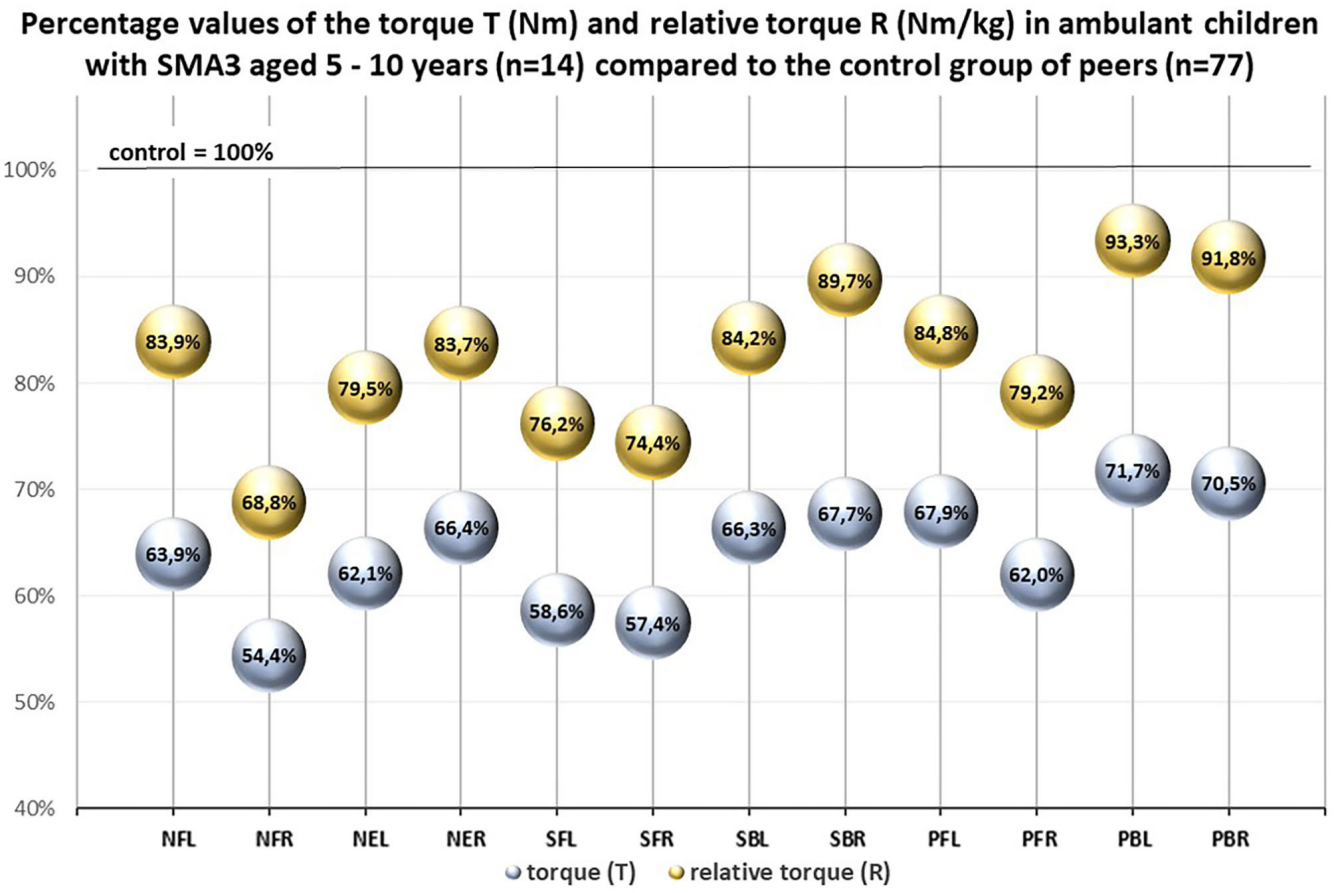
Percentage values of the torque T (Nm) and the relative torque R (Nm/ kg) in ambulant children with SMA 3 aged 5–10 years (*n* = 14) compared to the control group of peers (*n* = 77).

An additional comparison between ambulant children with SMA3 (*n* = 14) and the control group (*n* = 77) confirmed that ambulant children with SMA3 were significantly weaker than their healthy peers (Table 4). The percentages values of the relative torque R in this group calculated in relation to the control group were higher than the percentages values of the T coefficient (Figure 4).

## Discussion

In the presented study, an attempt was made at assessing neck and trunk muscle strength with the handheld digital muscle tester MICROFET2 in children and youth with different types of SMA and referring the obtained values to the norms. To date, no similar studies have been carried out. In the past, only limb muscle strength with the handheld tester in SMA patients was examined after previous measurement reliability assessment (2). Due to this, prior to the main measurements, the MICROFET2 device was assessed in the group of children with SMA and healthy controls, which proved high reliability of the measurements. Excellent reliability of the measurements with this device was revealed earlier in the research carried out in different populations (24, 25).

While planning the methodology of the study, it was discussed in what position children with SMA, particularly with SMA1 and SMA2, would be able to activate neck and trunk muscles because many of the untreated children were not able to perform movements resisting gravity. It was observed that side lying position made it possible to tone different groups of neck and trunk muscles even for the weakest children. Simultaneously, this position is used by patients with SMA in everyday functioning. Therefore, side lying position was used in the strength measurements and the methodology of testing the strength was adapted to the capabilities of patients with different types of SMA, which made it possible to compare these groups. Children from the age of five were qualified for the research as the initial measurement attempts revealed that from this age, children can cooperate, understand, and follow instructions well.

The manner of normalising the measurements also required discussion in order to minimise differences between the values of body mass and height in the participants with SMA and healthy controls. The muscle strength was measured parallel to the ground, not against gravity. Measuring the force with a dynamometer in these conditions required not only overcoming the participant's strength, but also counteracting body weight. This situation was more favourable for children with higher body weight. Therefore, we decided to use body weight to normalise the measurements. For this purpose, the values of strength in newtons were calculated as torques and then divided by body mass. In order to normalise other parameters applied in scientific studies, such as, lean body mass or body mass index would require taking into account the values of height. However, children and youth with SMA have contractures and spine deformities which make it difficult to measure height in a reliable way. Moreover, it was also taken into account that a similar manner of normalisation with body mass was applied in other studies on individuals with different types of muscular dystrophy (29).

During the analysis, the values of the torque T (Nm) and R coefficient (Nm/kg) were taken into consideration. According to the authors, R coefficient is more practical and should be included in the analysis and clinical conclusions. R coefficient made it possible to calculate strength per 1 kg of body mass, which allowed us “give equal chances” to all participants. The use of the relative R torque was beneficial for all tested SMA groups, because it resulted in an increase in the percentage of force measurements in relation to the control group. R coefficient analysis revealed more significant differences between the SMA subgroups. Moreover, the comparison of the values of R coefficient in SMA3 group and control group revealed fewer significant differences than in the case of the comparison regarding T coefficient. Taking into account the fact that the examined group included 15 children with SMA3 who, similarly to healthy children, could walk, and perform movements against gravity, these results should be treated as more feasible. The correlation between muscle strength and anthropometric factors in children with SMA should be analysed in future studies, among others due to the need for the assessment of the effectiveness of the presently applied medications.

The results showed that neck and trunk muscle strength in persons with SMA is significantly lower than among their healthy peers. The values of R coefficient regarding the area of head and trunk and taking into account body mass of the participants ranged from 42.6 to 68.4% in SMA1 group, from 54.8 to 75.4% in SMA2 group, and from 76.7 to 94.8% in SMA3 group with reference to the values of strength in the control group (Figure 3).

Participants with SMA1 and SMA2 turned out to be significantly weaker than their healthy counterparts in all the measurements. Children with SMA3 aged 5–16 obtained lower values of the torque T measurements, but at the same time, the analysis of the relative torque R showed no significant differences in several measurements compared to the control group. Analysis of the strength values in the group of participants with SMA3 aged 5–10 and healthy peers also showed a decrease in strength in children with SMA3, with the exception of a few parameters. Thus, it has been shown that children with SMA, regardless of type, show lower values of neck and trunk muscle strength than their healthy peers.

It is difficult to refer the obtained results to the findings of other studies due to the fact that to date, neck and trunk muscle strength has not been measured according to the presented methodology. In general, our results confirm the data provided by other researchers who, basing on limb muscle strength measurements, revealed that individuals with SMA are weaker than healthy persons (8, 10, 15). Kroksmark et al. (15) described decreased isometric muscle strength in patients with SMA2 and SMA3 with regard to the norms. In turn, the research by Merlini et al. (8) revealed that limb muscle strength in SMA patients is reduced and equals ~20% of the norm for the particular age and gender group. Febrer et al. (10) compared the values of maximum voluntary isometric contraction in four muscle groups obtained by SMA2 and SMA3 patients compared to the controls. It was proved that limb muscle strength in SMA patients is lower than in the control group, while the quadriceps muscle proved to be the weakest muscle. Other studies showed that the weakest muscles in SMA patients include triceps, deltoid, iliopsoas and quadriceps (14). Granger et al. (18) showed that maximum bite forces in SMA patients were reduced to 50% compared to a healthy controls. Masticatory muscle weakness relative to normal was also observed by Kruse et al. (19). None of the researchers described the objective values of neck and trunk muscle strength in people with SMA and compared them to the norms. The lack of studies in this area may result from the fact that neck and trunk strength measurement methods applied in other populations are not adapted to the functional state of SMA patients.

Our study revealed the correlation between the strength of neck and trunk muscles and the ability to change the position from lying on one's back to side lying unassisted. Participants with SMA2 and SMA3 who independently turned to the left and right side obtained higher values of relative torques than children with SMA1 and SMA2 who could not perform this activity. All the children with SMA1 and the majority of children with SMA2 needed help while changing a position at night. Children with SMA3 who obtained significantly higher values of strength than SMA1 and SMA2 patients did not have a problem with turning to one's side and less often required help at night compared to children with SMA1 and SMA2; however, they asked for help at night more often than healthy children. In turn, children with SMA1 needed help at night significantly more often than children with SMA2. It confirms the findings of other researchers that children with SMA1 cannot turn independently (30), while patients with SMA2 and SMA3 need help in everyday functioning (31).

In the literature, in has been frequently highlighted that muscle strength in individuals with SMA correlates with motor function (8, 10–13, 15–17). Kroksmark et al. (15) examined correlations between the strength of selected muscles of the limb measured with a myometer and the performance of 20 movements in six children and adolescents with SMA2 and eight with SMA3. It was concluded that the muscle weakness affected motor function in all participants (15). While assessing muscle strength in the upper and lower limbs by a hand-held dynamometer, timed walking, arising from the floor and climbing steps, Merlini et al. (8) revealed correlation between motor function and muscle strength in SMA patients. When examining patients with SMA2 and SMA3, Kauffman et al. (11) noted a significant relationship between Hammersmith Functional Motor Scale Expanded and elbow flexion, knee extension, and knee flexion strength (11). Werlauff et al. (12) observed that the decrease in muscle strength was reflected in deterioration of upper limb function in SMA2 and SMA3 patients. In the study by Seferian et al. (13), the distal force measurements of the upper limbs, were correlated to functional scales. Chabanon et al. (17) revealed that in patients with SMA2 and SMA3, there exists a correlation between grip and pinch strength and Motor Function Measure score. In the study on SMA2 and SMA3 patients, Febrer et al. noted differences in the strength of selected limb muscles of walkers and non-walkers. Non-walkers achieved lower values of strength (10). Montes et al. (16) described the correlation between knee flexor and hip abductor strength fatigue during gait in participants with SMA. Peteers et al. (21) revealed that while sitting, patients with SMA2 and SMA3 move their trunk to a limited extent in all directions, while the activity of back and abdominal muscles is lower than in the control group. This research also revealed that abdominal muscles are less engaged than back muscles. In our study, the strength of muscles performing the trunk movement to the back among SMA3 patients did not always differ from the strength of healthy children, while the strength of muscles active in moving forward was significantly lower. It can be concluded that the results of our study confirm the observation of other authors, indicating the relationship between the muscle strength and functional abilities of patients with SMA. In the future, however, the correlation between the strength of the neck and trunk muscles and various activities included in functional scales should be examined.

It may be noted that the studies analysing muscle strength in SMA2 and SMA3 patients paid less attention to SMA1 patients. In our study, the percentage values were higher (from a few to a dozen or so percent) in SMA2 group than among the SMA1 participants, however, due to the small size of the SMA1 group, these results should be treated with caution. It is worth noting many significant correlations between the measurements of neck and trunk muscle strength in SMA2 patients, SMA3 patients and healthy controls, as opposed to individuals with SMA1 whose correlations between the measurements in the area of head, upper trunk and lower trunk were not always significant.

The decreased neck and trunk muscle strength in children and adolescents with SMA may suggest the need for strengthening exercises, particularly due to the fact that the study including healthy participants indicates that trunk muscles are active during numerous everyday activities. While investigating the groups of healthy individuals, several research teams revealed that abdominal muscles are responsible for stabilising the trunk during movements of upper and lower limbs in a sitting and standing position (32–35) and are active while breathing (36, 37). In the study on healthy children and children with developmental coordination disorders, Kane and Bartel assessed the activation of e.g., transversus abdominis muscle or external and internal oblique muscles with surface electromyography during, inter alia, kicking a ball, climbing stairs, and single leg balance test. A significantly lower activation of abdominis muscles was revealed in children with developmental coordination disorders (35). Klemetti at al. claimed that abdominal and back muscles play a significant role during gait by controlling movements in the sagittal and coronal planes (38). It was also concluded that decreased trunk muscle strength may lead to pain (39) and postural disorders in healthy adolescents (40). In the study directed at isokinetic muscle strength assessment, Bernard et al. noted that the participants aged 14–16 with low back pain had weaker extensors and stronger flexors of the trunk compared to their peers without any health problems (39). Barczyk and Pawelec et al. found that decreased trunk flexor muscle strength accompanies body posture disorders in children aged 10–11 (40). The above studies revealed the need for trunk muscle training and may serve as an idea for scientific projects on SMA patients.

The presented study has some limitations. The device and methods applied in this study have not been used in neck and trunk muscle measurements to date. Simultaneously, it is difficult to conclude whether strength values obtained by children can be seen as maximal. The results may include inaccuracies due to the difficulties cooperating with children who sometimes show the lack of concentration, fatigue, impatience, or aversion. The measurement values can also be affected by the strength of the researcher which is considerably higher than the child's strength. In the past, it was pointed to the potential correlation between the strength of the researcher and the measurements of strength in the participants (41). The debatable part of our study is including the weakest children with the types 1 and 2 to the project. The authors realise that the structural changes in the muscles (42) may influence the strength measurements in these participants. However, the obtained results may be useful in future studies involving children receiving pharmacological treatment. Accurate information on the duration of daily non-invasive ventilation in subjects was not collected during the study. Respiratory functions and time of ventilation support may be related to the strength of the examined muscles, and it is worth considering this potential relationship in future studies. This study involved nine children with SMA1 type who used non-invasive ventilation at night and periodically during the day. Taking into account the fact that many children with SMA1 type, due to the severe course of the disease, use continuous respiratory ventilation, the participants with type SMA1 in our study should be considered unique. The small size of the SMA1 group and the milder course of the disease could influenced the obtained results, especially the lack of differences between SMA1 and SMA2 groups. An additional factor that may increase the differentiation between the SMA groups and the control group is the age difference. Although, the analysis carried out among children aged 5–16 did not show significant differences between the age of children in the SMA1, SMA2, and control groups, participants with SMA were younger than the controls. Similarly, participants aged 5–10 with type 3 SMA were several months younger than healthy children. In our study, strength values were related to the ability to change position from supine to side lying. The use of reliable functional scales recommended for patients with SMA would enable a much wider analysis of the relationship between the strength and motor function. Additionally, it should be investigated in the future which muscles of the neck and trunk are active when taking particular strength measurements.

The study broadens the knowledge regarding the natural course of the disease, with particular focus on the strength of neck and trunk muscles in non-pharmacologically treated SMA patients. This knowledge seems to be significant due to the fact that in a short period of time, an access to untreated patients will be hampered owing to the introduction of pharmacological treatment. Simultaneously, it is worth noting that head movements and trunk rotations are taken into account in functional scales used to assess patients with SMA (43–50). According to International Classification of Functioning, Disability and Health, the examination should include an assessment of body structures and functions, activity and participation. We believe that the measurements of the strength of neck and trunk muscles could be used as an additional type of assessment of the applied pharmacological treatment and other therapeutic procedures. An improvement in neck and trunk muscle strength may lead to an increase in the number of points in functional scales, which confirms the effectiveness of the treatment. The assessment of muscle strength will also make it possible to perform a detailed analysis of correlations between functional capabilities of children and strength of particular groups of muscles.

To sum up, it may be concluded that the measurements of neck and trunk muscle strength with a digital handheld muscle tester are a reliable way of testing and could be used to assess children and youth with SMA. Body mass is a significant factor affecting the measurements of neck and trunk muscle strength. The presented results scientifically confirmed the theory based on clinical observations that neck and trunk muscle strength in SMA children not treated pharmacologically is lower than in healthy controls and depends on disease type. Patients with SMA are not a uniform group in terms of neck and trunk muscle strength assessment criterion, which makes it difficult to plan the repeatable and systematic research methodology. In the future, the effect of pharmacological treatment on the strength of the neck/trunk muscles, and the relationship between muscle strength and motor skills should be investigated.

## Data Availability Statement

The datasets generated for this study are available on request to the corresponding author.

## Ethics Statement

The studies involving human participants were reviewed and approved by Senate Research Ethics Committee, Józef Piłsudski University of Physical Education, Warsaw. Written informed consent to participate in this study was provided by the participants' legal guardian/next of kin.

## Author Contributions

AS study design, literature research, clinical examination of the participants, supervision of statistical analysis, and manuscript writing. TO clinical examination of the participants, collecting data, and literature research. WR preparation of databases and statistical analysis. AW data interpretation and critical assessment of the integrity of the manuscript. All authors approved the final manuscript.

## Conflict of Interest

The authors declare that the research was conducted in the absence of any commercial or financial relationships that could be construed as a potential conflict of interest.

## Abbreviations

SMA (1, 2, 3): spinal muscular atrophy (type 1, 2, 3)
NFL: neck flexion in side lying on the left
NFR: neck flexion in side lying on the right
NEL: neck extension in side lying on the left
NER: neck extension in side lying on the right
SFL: right scapula forward in side lying on the left
SFR: left scapula forward in side lying on the right
SBL: right scapula backward in side lying on the left
SBR: left scapula backward in side lying on the right
PFL: right part of pelvis forward in side lying on the left
PFR: left part of pelvis forward in side lying on the right
PBL: right part of pelvis backward in side lying on the left
PBR: left part of pelvis backward in side lying on the right
F: force measurement
a: moment arm
T: the torque, calculated by multiplying the force measurement (F) by the moment arm from the axis of motion (r)
BM: body mass
R: the relative torque, calculated by dividing the value of the torque (T) by the body mass (BM)
ICC: interclass correlation coefficient
r: correlation coefficient.
